# Overexpression of a NAC transcription factor delays leaf senescence and increases grain nitrogen concentration in wheat

**DOI:** 10.1111/plb.12296

**Published:** 2015-01-16

**Authors:** D. Zhao, A. P. Derkx, D.‐C. Liu, P. Buchner, M. J. Hawkesford

**Affiliations:** ^1^ Northwest Institute of Plateau BiologyChinese Academy of SciencesXi'ningQing'haiChina; ^2^ Plant Biology and Crop Science DepartmentRothamsted ResearchHarpendenUK; ^3^ University of Chinese Academy of SciencesBeijingChina

**Keywords:** Grain protein deviation, NAC gene family, nitrogen remobilisation, rubisco, Triticum aestivum, yield

## Abstract

Increasing the duration of leaf photosynthesis during grain filling using slow‐senescing functional stay‐green phenotypes is a possible route for increasing grain yields in wheat (*Triticum aestivum* L.). However, delayed senescence may negatively affect nutrient remobilisation and hence reduce grain protein concentrations and grain quality. A novel NAC1‐type transcription factor (hereafter TaNAC‐S) was identified in wheat, with gene expression located primarily in leaf/sheath tissues, which decreased during post‐anthesis leaf senescence. Expression of TaNAC‐S in the second leaf correlated with delayed senescence in two doubled‐haploid lines of an Avalon × Cadenza population (lines 112 and 181), which were distinct for leaf senescence. Transgenic wheat plants overexpressing TaNAC‐S resulted in delayed leaf senescence (stay‐green phenotype). Grain yield, aboveground biomass, harvest index and total grain N content were unaffected, but NAC over‐expressing lines had higher grain N concentrations at similar grain yields compared to non‐transgenic controls. These results indicate that TaNAC‐S is a negative regulator of leaf senescence, and that delayed leaf senescence may lead not only to increased grain yields but also to increased grain protein concentrations.

## Introduction

The growing world population requires an increase in crop production. The use of semi‐dwarfing genes during the Green Revolution led to dramatic increases in wheat yields in the last century (Hawkesford 2014). For further improvement of yields to fulfil growing demand, new targets and strategies must be identified. One possible route to increase crop yield is to extend the duration of photosynthesis (Richards 2000). This may be achieved by delaying leaf senescence during grain filling and creating a functional stay‐green phenotype (Thomas & Howarth 2000). Delayed leaf senescence has been reported to increase grain yield in wheat (Spano *et al*. 2003; Gong *et al*. 2005; Luo *et al*. 2006; Christopher *et al*. 2008; Chen *et al*. 2010), presumably because of prolonged photosynthetic duration.

The recycling of nutrients, specifically nitrogen (N), into developing tissues such as grain is considered the main function of leaf senescence (Gregersen *et al*. 2008). The proportion of grain N in wheat that originates from remobilisation of N taken up before anthesis is estimated as >70% (Kichey *et al*. 2007). Generally, greenness and total protein content of leaves during senescence are well correlated (Thomas *et al*. 2002). Patterns of leaf senescence should therefore correspond to N remobilisation from the leaves. A delay in leaf senescence would result in a delay in N remobilisation and have a negative impact on protein deposition to the grain, thus reducing grain quality.

A locus associated with early leaf senescence and increased grain protein content (GPC) was identified on chromosome 6B of wild emmer wheat *(Triticum turgidum* subsp. *dicoccoides)*. Recombinant wheat with this *Gpc‐B1* locus displayed early leaf senescence and improved remobilisation of amino acids from the flag leaf, thereby increasing GPC without reducing grain yield (Kade *et al*. 2005; Uauy *et al*. 2006b). Uauy *et al*. (2006a) identified a NAM, ATAF 1,2 and CUC2 (NAC) transcription factor, *NAM‐B1*, as the gene responsible for the *Gpc‐B1* phenotype. RNAi knockdown of *NAM‐B1* resulted in delayed leaf senescence, reduced N remobilisation from the leaves and consequently a lower GPC (Uauy *et al*. 2006a; Waters *et al*. 2009).

Members of the NAC transcription factor family have been shown to be involved in developmental and physiological processes, such as embryo and shoot meristem development, lateral root formation, auxin signalling, defence, abiotic stress responses and senescence (Olsen *et al*. 2005; Nakashima *et al*. 2012; Nuruzzaman *et al*. 2012). In addition to wheat *NAM‐B1*, senescence‐associated NAC transcription factors have been described in *Arabidopsis thaliana* (Guo & Gan 2006; Yoon *et al*. 2008; Kim *et al*. 2009; Balazadeh *et al*. 2011; Yang *et al*. 2011; Lee *et al*. 2012; Wu *et al*. 2012), rice (*Oryza sativa*; Sperotto *et al*. 2009), bamboo (*Bambusa emeiensis*; Chen *et al*. 2011) and barley (Christiansen & Gregersen 2014). The majority of these genes show increased expression during leaf senescence and are therefore positive regulators of senescence; only one gene in *Arabidopsis* and three genes in barley have been shown to be potential negative regulators of leaf senescence (Wu *et al*. 2012; Christiansen & Gregersen 2014).

In this study TaNAC‐S, a novel senescence‐associated NAC transcription factor showing decreased leaf expression during leaf senescence, was identified and expression profiles were analysed with respect to tissue specificity and post‐anthesis leaf developmental pattern. Furthermore, the influence of overexpressing the TaNAC‐S gene on leaf senescence, grain yield and N content was studied.

## Material and Methods

### Plant material

#### Field trial

Two Avalon × Cadenza doubled haploid wheat lines (lines 112 and 181), selected for contrasting canopy senescence, were grown in a field trial at Rothamsted Research (Harpenden, UK) in 2010/11 as part of the Defra‐funded Wheat Genetic Improvement Network (WGIN) project. The population of doubled‐haploid individuals derived from F_1_ progeny of a cross between cv. Avalon × Cadenza was developed by Clare Ellerbrook, Liz Sayers and the late Tony Worland (John Innes Centre) as part of a Defra‐funded project led by ADAS. The parents were originally chosen (for contrasting canopy architecture traits) by Steve Parker (CSL), Tony Worland and Darren Lovell (Rothamsted Research). The field trial was grown at four N fertiliser levels (0, 100, 200 and 350 kg N·ha^−1^) in three randomised replicate blocks at the Summerdells I and II fields at Rothamsted Research. Senescence measurements were performed at anthesis and weekly thereafter until complete senescence at 8 weeks post‐anthesis (wpa). For gene expression and N analyses, replicate flag leaf and second leaf samples (10 leaves from 10 main shoots per sample) were harvested each week, starting from anthesis, and immediately frozen in liquid N.

#### Production of transgenic wheat plants

The cDNA containing the full open‐reading frame of the senescence‐associated wheat NAC‐S was isolated with PCR using the sequence information of HM037184 from leaf RNA of wheat cv. Cadenza. After sequencing, the wheat NAC‐S DNA fragment was inserted between the rice tungro bacilliform virus (RTBV) promoter (Bhattacharyya‐Pakrasi *et al*. 1993) and the 35S termination signal into a modified pBract302 plasmid, in which the ubiquitin promoter‐nos terminator cassette was replaced by the RTBV‐promoter‐TaNAC‐S‐35S‐terminator construct. The plasmid contained the *NPT1* kanamycin resistance gene for selection of bacteria and the *BAR* gene conferring phosphinothricin resistance under control of a ubiquitin promoter to allow for plant selection. The construct was introduced into wheat cv. Cadenza using particle bombardment of wheat embryos (as described in Sparks & Jones 2009). Eight independent transgenic plants were generated, namely NAC R1, R2, …R8. Plants were self‐fertilised to obtain T_1_ and T_2_ plants. Genomic DNA from T_1_ and T_2_ plants was extracted in the seedling stage with the CTAB method (Lin *et al*. 2001) and transgenic lines were screened using PCR with the specific primers (Table 1). Homozygosity and copy number of T_2_ plants were determined by IDna Genetics Ltd. (Norwich, UK). Homozygous T_2_ plants were self‐fertilised to obtain T_3_ plants.

**Table 1:** Primers used for SYBR green real‐time PCR expression analysis

gene	accession number	forward primer	reverse primer
TaActin3 like	TC234027	GACGCACAACAGGTATCGTGT TG	GACGCACAACAGGTATCGTGT TG
TaNAC‐S	TA67563_4565; TC368951	GAGCTGGACCTTCCGACTATGC GCCAGGAGAACGGCTTGTCACA	CACTTGCTCGATGGTGTTCATC CCACCACACTTCAACTAGTAGC
TaNAM‐B1	HQ872050	CTA CAA GAA GAT CAA CAA GGC CGC	TCC ACG GAG TCC TCG CAC TC
TaRubiscoSSU8	TC263601	GGT GGA GGA GGT CAA GAA GGA G	MGT CGT GAG TGA GCT GTT TAG GC
TaRubiscoLSU	AY328025	ACCATTTATGCGCTGGAGAGACC	CAAGTAATGCCCCTTGATTTCACCG
TaSAG12	AB267407	CACTGTCAGTGAAGGCTCAGAG	GTCGTGATGCAAATGTTTACGCG

#### Glasshouse‐grown plant material

Canopy plant tissues from the root, stem, sheath of leaf 3 (third leaf from the shoot tip), sheath of leaf 2, sheath of flag leaf (leaf 1), leaf 3, leaf 2 and flag leaf were harvested from pot‐grown wheat plants (cv. Cadenza). All tissues were harvested at anthesis.

To analyse the influence of overexpression of the TaNAC‐S transgene driven by the RTBV promoter, three glasshouse experiments were conducted under controlled environment conditions (20 °C day/15 °C night temperatures with a 16‐h photoperiod), one to study gene expression and two for N and yield measurements. For gene expression and chlorophyll analysis, three homozygous transgenic lines, NAC‐R5, ‐R6 and ‐R8, and control Cadenza were grown in a randomised block design with three replicates. The top three leaves of the main shoot were sampled for gene expression/chlorophyll analyses from anthesis to 7 wpa.

For N measurement, one experiment involved three positive plants, NAC‐R4, ‐R5 and ‐R8 and control Cadenza, in a complete randomised block design with four replicates. The second experiment contained five transgenic lines, NAC‐R2, ‐R3, ‐R5, ‐R6 and ‐R8, and both non‐transformed and transformed control Cadenza, in a randomised block design with four replicates.

### Chlorophyll measurement

Samples were ground to a fine powder under liquid N and freeze‐dried. Chlorophyll was extracted twice with 80% and once with 50% ethanol. The extracts were combined and mixed and the absorbance of the extracts at 645 nm and 665 nm measured with a Gemini XPS Fluorescence Microplate Reader (Molecular Devices, Sunnyvale, CA, USA). Finally, chlorophyll content was calculated based on Wintermans & de Mots (1965).

### Nitrogen analysis

The N concentrations of oven‐dried (80 °C), milled grain and straw material was analysed using either a CNS 2000 or a TruSpec Combustion Analyser (both from LECO Corp., St. Joseph, MI, USA) with the Dumas combustion method (Dumas 1831).

### Gene expression analysis

Gene expression was analysed using quantitative (different issues) or relative real‐time PCR (time‐course experiments). Total RNA was extracted using a modified hot phenol extraction method based on Verwoerd *et al*. (1989): prior to LiCl precipitation, an additional chloroform/isoamyl alcohol (IAA) extraction step was integrated and genomic DNA contamination removed through DNase treatment including a phenol/chloroform/IAA extraction and a final ethanol/Na acetate precipitation. cDNA was synthesised from 2 μg total RNA and oligo‐dT‐adapter primer in a 20‐μl reaction based on a Superscript III Reverse Transcriptase standard protocol (Invitrogen, Carlsbad, CA, USA), including 2 h synthesis time to ensure complete full‐length cDNA synthesis. Real‐time PCR was performed using the Applied Biosystems 7500 Real‐Time PCR System and SYBR Green JumpStart Taq ReadyMix (Sigma‐Aldrich, St. Louis, MO, USA). The 25‐μl reactions contained 1 μl cDNA and 250 nm of each primer (Table 1).

Relative gene expression of the target genes was calculated using the NRQ method (Rieu & Powers 2009), with normalisation of a constitutive control wheat gene, the Actin 3 gene. Wheat Actin 3 gene was shown to have the best linear constitutive performance at all post‐anthesis time points/replicates for all tested control genes. This was verified with wheat post‐anthesis leaf material (unpublished data) using SYBR‐green real‐time PCR and comparing so‐called constitutive genes, α‐tubulin, a cyclin, 18S ribosomal, cyclophilin and Actin 3 gene. For accuracy of the analysis, the mean primer efficiency was estimated using the linear phase of all individual reaction amplification curves (Ramakers *et al*. 2003), calculated using the LinRegPCR package (Tuomi *et al*. 2010). The normalised relative quantity (NRQ) of expression was calculated in relation to the CT values, the primer efficiency (E) of the target gene (X) and the normalising reference gene (N), as normalised relative expression (NRE) based on Rieu & Powers (2009):

(1) $$\text{NRE}={\left(E_{X}\right)}^{-\mathrm{CT},\;X}/{\left(E_{N}\right)}^{-\mathrm{CT},\;N}$$

For absolute expression analysis, TaNAC‐S specific DNA fragments were PCR‐amplified from cDNA and cloned into pGEM‐Te vector (Promega, Southampton, UK). Quantitative real‐time SYBR green PCR expression analysis was performed including a standard dilution series of TaNAC plasmid‐PCR fragments in triplicate. Based on the molecular weight of plasmid and PCR fragment, the mRNA copy number per microlitre cDNA was calculated after actin normalisation of CT values.

### Phylogenetic analysis

Protein sequences of all known rice (*Oryza sativa* ssp. *Japonica*) NAC genes were downloaded from DRTF (Database of Rice Transcription Factors), while known wheat NAC genes and known senescence‐related NAC genes from other plant species were downloaded from the UniProtKB database. The NAC domain protein sequences were aligned using MEGA5 software with the built‐in ClustalW algorithm (Larkin *et al*. 2007). Phylogenetic trees were constructed using the neighbour‐joining method in MEGA5 (Tamura *et al*. 2011). The stability of the nodes was tested using bootstrap analysis with 1000 replicates implemented in MEGA5.

### Statistical analysis

Analysis of variance (anova) and linear regression with groups were performed using GenStat software (Payne *et al*. 2009).

## Results

### The TaNAC‐S transcription factor gene/protein sequence and phylogenetic analysis

A NAC transcription factor was identified in a transcriptomics study (Affymetrix wheat1 gene chip: Ta.16423.1.S1_at) of six wheat varieties grown with several levels of N (Hawkesford & Howarth 2010). Expression of the transcription factor showed a strong positive correlation with leaf N concentration, and was suggested as a candidate gene that might be involved in leaf senescence and N remobilisation during grain filling (J.R. Howarth and M.J. Hawkesford, unpublished). The full‐length coding sequence, including 5′ and 3′ non‐coding regions, was obtained from the HM037184 sequence accession homologous to CK208366, and named TaNAC‐S.

**Figure 1:**
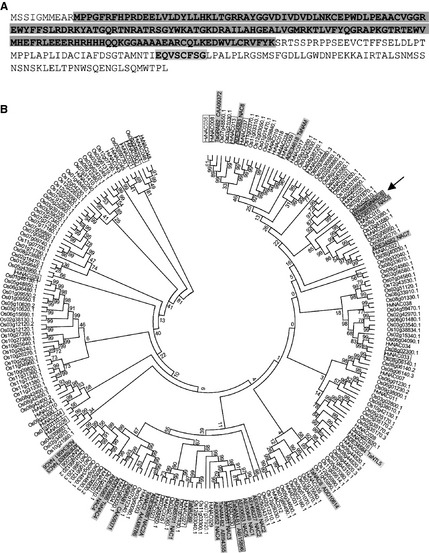
Sequence and phylogenetic analyses of the NAC gene. A: Protein sequence of the NAC gene. The NAC domain and the NAC1/NAM group‐specific motif are highlighted in grey. B: Phylogenetic analysis of the novel wheat NAC gene (arrow, bold, squared, grey), known wheat NAC genes (grey), described barley NAC genes (Christiansen & Gregersen 2014; all senescence‐associated are squared) and all rice (ssp. *Japonica*) NAC genes. Amino acids within the NAC domain were aligned in MEGA5 using the ClustalW algorithm (Larkin *et al*. 2007). The phylogenetic tree was constructed using the neighbour‐joining method (Tamura *et al*. 2011). Bootstrap values of 1000 replicates are indicated at each node.

Ooka *et al*. (2003) identified five subdomains within the NAC domain, and 13 motifs in the transcriptional activation region (TAR). The wheat TaNAC‐S protein sequence contains all five subdomains, as well as a NAC1/NAM group‐specific motif (Fig. 1A). To determine the relationship with previously described NAC genes, a phylogenetic tree of NAC protein sequences was constructed (Fig. 1B). The tree encompasses currently described senescence‐associated NAC genes from several plant species (*Arabidopsis*, bamboo, rice, tomato and wheat), all rice (ssp. Japonica) NAC proteins and all described wheat NAC proteins to date. The wheat TaNAC‐S gene did not show a close relationship with any previously studied senescence‐associated NAC genes. The novel wheat NAC protein closely resembled another wheat NAC protein, ADG85703, which has an identical NAC domain but differs in three residues outside the domain (data not shown). The closest related rice NAC gene was Os08g10080, named ONAC104 by Fang *et al*. (2008), which is a NAC1‐type NAC transcription factor (Fang *et al*. 2008; Nuruzzaman *et al*. 2010). To date, biological functions of ADG85703 and ONAC104 have not been described. In summary, the sequence and phylogenetic analyses suggested the wheat TaNAC‐S gene is a transcription factor of the NAC1‐type that has not been previously characterised.

### Expression pattern of TaNAC‐S transcription factor

**Figure 2:**
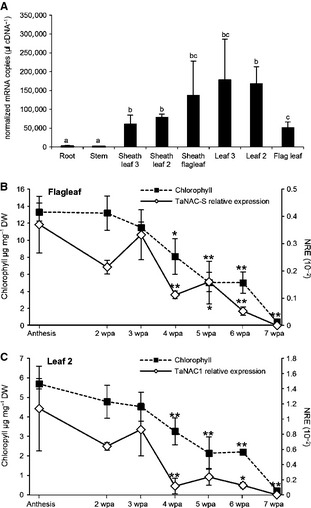
A: Absolute real‐time PCR transcript analysis of TaNAC‐S in different canopy tissues of wheat. Each value is normalised against the control gene, as described in the text. Each bar represents mean ± SE of at least three biological replicates. Different letters indicate differences significant at 0.05 level. B: Relative TaNAC‐S gene expression analysis in relation to post‐anthesis chlorophyll content/senescence in the flag leaf and C: leaf 3 of wheat cv. Cadenza determined with SYBR‐Green real‐time PCR. NRE: Constitutive control gene Normalised Relative Expression as described in text; values are means ± SEM (n = 3). **P <* *0.01, *P <* *0.05 significantly different changes in time compared to anthesis; wpa, weeks post‐anthesis.

Expression of the TaNAC‐S gene in the different wheat vegetative components at anthesis was leaf 3> leaf 2> sheath of flag leaf > sheath leaf 2 and leaf 3> flag leaf, and very low in stem and root tissues (Fig. 2A). The expression of TaNAC‐S in leaves after anthesis showed a correlation with senescence (Fig. 2A,B). In both flag leaves and leaf 2, the relative gene expression followed the reduction in chlorophyll.

To examine expression in field‐grown wheat and explore possible genetic variation of TaNAC‐S gene expression in wheat, expression was analysed in two Avalon × Cadenza doubled‐haploid lines grown in field trials that appeared to show contrasting senescence patterns (P. Buchner and M.J. Hawkesford, unpublished observations). The senescence pattern indicated as leaf chlorophyll content of line 181 was more advanced than that of line 112 at anthesis and in the post‐anthesis period, particularly for the flag leaf. For leaf 2, chlorophyll content only decreased in line 181 compared to 112 at 6 wpa.

**Figure 3:**
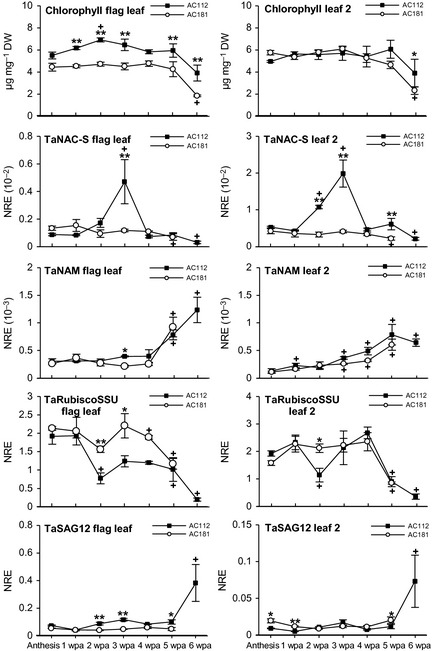
Post‐anthesis chlorophyll content and mRNA abundance of TaNAC‐S, TaNAM‐B1, Rubisco SSU and TaSAG12 in flag leaf and leaf 2 of field‐grown double‐haploid lines 112 and 181. NRE: Constitutive control gene Normalised Relative Expression; *P ≤* *0.05, **P ≤* *0.01 significant differences between AC112 and AC181; ^+^ *P *≤* *0.05 significant changes to anthesis; values are mean ± SEM (n = 3).

The TaNAC‐S gene expression pattern differed between the two wheat lines. In general, expression was fivefold lower in flag leaves compared to leaf 2 in both lines. Line 112 showed a similar post‐anthesis expression pattern in both leaf types having increasing transcript levels, with a peak at 3 wpa, which subsequently decreased to almost the initial level at 4 wpa (Fig. 3), with a further significant reduction of expression at 6 wpa. The expression of TaNAC‐S in flag leaves and leaf 2 of line 181 was quite distinct, with no equivalent peak of expression occurring. To compare the expression to other genes known to be influenced by senescence in wheat, the expression patterns of the small subunit (SSU) of Rubisco, the senescence‐associated wheat gene TaSAG12, coding for a C1A cysteine protease orthologous to the senescence associated *Arabidopsis* SAG12 gene (Noh & Amasino 1999), and the wheat NAC transcription factor TaNAM‐B1 (Uauy *et al*. 2006a; Waters *et al*. 2009) were analysed in leaves of lines 112 and 181 (Fig. 3). As previously described (Uauy *et al*. 2006a,b), the expression of TaNAM‐B1 increased with increasing leaf senescence in the flag leaf and leaf 2 in both DH lines. Contrasting expression of Rubisco SSU expression in the two lines (Fig. 3) was observed, particularly for the flag leaf; higher expression occurred in the flag leaf of line 181 compared to line 112 between 2 and 4 wpa. Rubisco SSU expression decreased from 4 to 6 wpa (Fig. 3). TaSAG12 shows increased expression with increasing senescence in wheat (Buchner & Hawkesford 2014). In the flag leaf of line 112, a slight increase in expression compared to anthesis was seen between 3 and 5 wpa, with a more than fivefold increase of expression by 6 wpa. A similar increase was found in leaf 2 of line 112. There was no change in TaSAG12 expression in flag leaves and leaf 2 of line 181.

### Overexpression of the TaNAC‐S transcription factor delays leaf senescence

**Figure 4:**
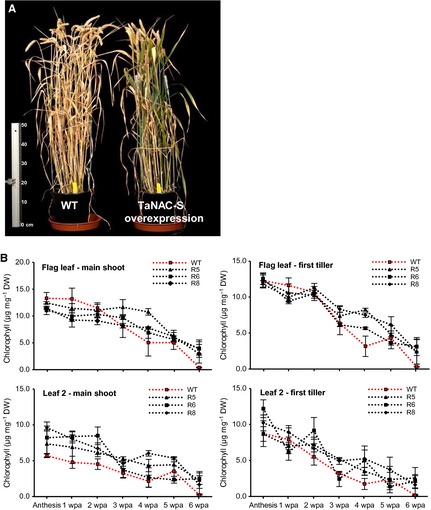
A: Representative plants of transgenic and control show difference in greenness at 8 weeks post‐anthesis (126 days after germination). B: Chlorophyll content of flag leaf and leaf 2 on main shoot and first tiller of wild type control (in red) and transgenic TaNAC‐S overexpression lines from anthesis to 7 weeks post‐anthesis. Values are mean ± SEM (n = 3).

Overexpression of TaNAC‐S in transgenic wheat results in a delayed post‐anthesis senescence phenotype in comparison to non‐transgenic Cadenza wild type and negative transformed control wheat plants (Fig. 4). The wild type and control wheat plants had mostly completely senesced by 8 wpa. In contrast, canopies of the analysed TaNAC‐S overexpressing plants were still partially green at 8 wpa (Fig. 4A). The post‐anthesis chlorophyll content in flag leaves and leaf 2 on the main shoot and first tiller was similar in TaNAC‐S overexpressing lines in comparison to the Cadenza wild type, apart from a strong decrease of chlorophyll content to nearly zero in wild‐type plants between 6 and 7 wpa, which was not found in the TaNAC‐S overexpressing lines (Fig. 4B).

### Gene expression in transgenic wheat

**Figure 5:**
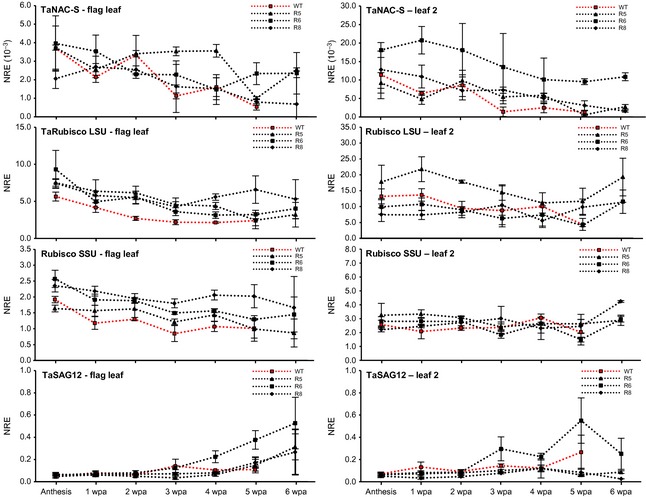
Post‐anthesis mRNA abundance of TaNAC‐S, Rubisco LSU, Rubisco SSU and TaSAG12 in flag leaves and leaf 2 of TaNAC‐S overexpressing transgenic wheat lines R5, R6 and R8 and non‐transgenic wheat cv. Cadenza wild‐type controls (in red). NRE: Constitutive control gene Normalised Relative Expression; values are mean ± SEM (n = 3).

The aim of the overexpression of TaNAC‐S under control of the rice tungro bacilliform virus (RTBV) promoter (Bhattacharyya‐Pakrasi *et al*. 1993) was to achieve leaf‐targeted expression; the localisation was confirmed in RTBV promoter GUS studies (unpublished). As found in other expression studies, expression of TaNAC‐S in overexpressing lines and control plants was higher in leaf 2 than in the flag leaf (compare Figs 2, 3 and 5). There was no available RNA of control plants for gene expression analysis due to full senescence at 7 wpa. In the flag leaf and particularly in leaf 2, significantly higher gene expression of TaNAC‐S was found for most transgenic lines compared to non‐transgenic control plants at all post‐anthesis time points (Fig. 5). Activity of the RTBV promoter seemed to be much lower than reported for other well‐known constitutive promoters such as the 35S or rice actin promoters. Throughout the post‐anthesis senescence period there was a significant continuous reduction of TaNAC‐S gene expression in control plants from 4 wpa onwards until complete senescence, which was less pronounced in the transgenic lines. In the flag leaf, TaNAC‐S expression was generally highest in line R5; however, in the flag leaf, line R6 had much higher TaNAC‐S expression, with lines R5 and R8 also having significantly higher TaNAC‐S gene expression from 3 and 4 wpa onwards compared to the non‐transgenic control plants. Between anthesis and 6 wpa there was a 10‐fold reduction of TaNAC‐S expression in flag leaves of control plants. For transgenic line R6, there was no significant change in post‐anthesis expression. There was a small significant reduction of TaNAC‐S expression in lines R5 and R8 between 4 and 7 wpa; however expression in R5 at 7 wpa was still almost fivefold higher compared to control expression at 6 wpa.

To verify the influence of TaNAC‐S overexpression on other genes, the expression patterns of the large and small subunits of the Rubisco (SSU/LSU) and the TaSAG12 cysteine protease were analysed. Gene expression of the Rubisco LSU in the flag leaf was significantly higher in TaNAC‐S overexpressing plants compared to control plants at almost all time points (Fig. 5). This was found in leaf 2 at only a few time points and only for line R5. Whilst there was a significant reduction of Rubisco LSU transcripts over the time course for flag leaves and leaf 2 of control plants, a similar pattern was found only in flag leaves for line R5.

The gene expression of Rubisco SSU did not change significantly between anthesis and 6–7 wpa for both flag leaf and leaf 2 for all transgenic lines and controls (Fig. 5). Whilst there was a clear significant increase of TaSAG12 gene expression over time in the flag leaf of the TaNAC‐S overexpressing lines, control plants did not show any increase in TaSAG12 expression, in spite of the more rapid senescence pattern (Fig. 5). The opposite was seen in leaf 2 for lines R5 and R8. In control plants, TaSAG12 expression increased at late senescence but there was no change of expression in lines R5 and R8. In line R6, there was increased expression from 3 to 5 wpa, which decreased at 6 wpa (Fig. 5).

### Grain yield and N content

There were no consistent differences in total N in the grain and grain percentage N between transgenic lines and controls (Table 2), while total straw N content and percentage N in straw of all the transgenic lines (except percentage N of straw in R2) was significantly higher than that of the control, suggesting that the delayed canopy senescence may have resulted in more N intercepted in straw and chaff of the transgenic lines. The N amount per grain of nearly all transgenic lines (except line R2) was significantly higher than that of the control, suggesting improved N uptake in the grain and remobilisation in the straw (Table 2).

**Table 2:** Total grain N (g), grain percentage N (%), total straw N (g), straw percentage N (%) and N per grain (mg) for overexpressing wheat lines and controls in a greenhouse experiment conducted in 2013. Data shown as mean ± SE; different letters indicate significant difference at 0.05 level, controls are non‐transformed wild type Cadenza, ‘trans control’ is transformed but negative for the transgene

lines	grain N (g)	grain N%	straw N (g)	straw N%	N per grain (mg)
NAC‐S R2	1.66 ± 0.26 a	3.10 ± 0.10 b	0.70 ± 0.12 a	1.16 ± 0.33 b	1.63 ± 0.18 b
NAC‐S R3	1.48 ± 0.20 a	2.76 ± 0.14 c	0.83 ± 0.04 a	1.31 ± 0.20 a	2.13 ± 0.31 ab
NAC‐S R5	1.28 ± 0.31 a	3.14 ± 0.12 b	0.78 ± 0.12 a	1.32 ± 0.23 a	2.61 ± 0.62 a
NAC‐S R6	1.33 ± 0.13 a	3.21 ± 0.14 b	0.70 ± 0.07 a	1.22 ± 0.06 a	2.47 ± 0.18 a
NAC‐S R8	1.05 ± 0.16 b	3.84 ± 0.22 a	0.93 ± 0.11 a	1.93 ± 0.15 a	2.73 ± 0.28 a
control	1.63 ± 0.10 a	2.60 ± 0.08 c	0.59 ± 0.06 b	0.88 ± 0.08 b	1.49 ± 0.07 b
trans control	1.59 ± 0.12 a	3.01 ± 0.22 b	0.59 ± 0.03 b	0.88 ± 0.05 b	1.39 ± 0.05 b

For further verification of whether the delayed senescence changes N remobilisation from straw to seed, N amount per grain was calculated by dividing total grain N by grain number, and significant differences were observed between lines (*P *<* *0.05). The N amount per grain of transgenic lines was 0.140–1.234 mg higher than in controls; this difference was not due to a grain size dilution effect since there was no significant difference in 1000 grain weight between transgenic lines and controls (data not shown). This result further confirmed the improved remobilisation from vegetative organs to growing seed in transgenic lines.

**Figure 6:**
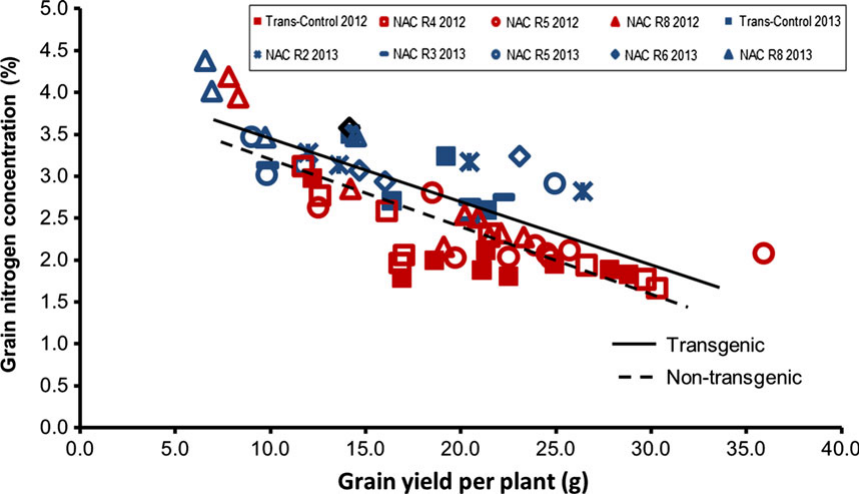
Linear regression between grain yield per plant (GY) and grain N concentration (GNC) of transgenic lines and controls. Data are from two independent glasshouse experiments; the first with three transgenic lines (R4, R5 and R8, conducted in 2012) and second with five lines (R2, R3, R5, R6 and R8, conducted in 2013), transformed but non‐transgene‐containing Cadenza as control. Regression equation for transgenic lines is y = −0.0755x + 4.1261 (R^2^ = 0.6119) and control lines is y = −0.0796x + 3.953 (R^2^ = 0.4237). The 2012 data are indicated in red and the 2013 data in blue.

Comparing grain N concentrations with grain yields for all lines gave an expected negative correlation (Fig. 6). A small displacement between the 2012 and 2013 data suggests that conditions were slightly different, despite the controlled glasshouse conditions. However, combining the data sets from the 2 years, and plotting separate regression lines for the transgenic and the control lines produced parallel regression lines. The correlation regression for the controls was lower than that for all transgenic lines, indicating a positive grain protein deviation (*i.e.,* higher than expected grain N content for a given yield) for the transgenic lines.

## Discussion

### The TaNAC‐S shows decreased expression following leaf senescence

In this study, a novel wheat NAC transcription factor, TaNAC‐S, was identified that displayed decreased expression during leaf senescence, and overexpression in a stay‐green phenotype, indicating the TaNAC‐S gene is a potential negative regulator of leaf senescence. NAC genes that are negative regulators of senescence appear to be rare: so far only the *Arabidopsis JUB1* gene has been unequivocally identified as a negative regulator of senescence (Wu *et al*. 2012). A further three NAC genes in barley (*HvNAC004, HvNAC042, HvNAC046*) with decreased expression at late senescence stages have been identified (Christiansen & Gregersen 2014), but are not orthologous to TaNAC‐S.

The NAC expression patterns during post‐anthesis leaf senescence of wheat were different in different genetic backgrounds, as observed in the double‐haploid lines, 112 and 181, suggesting control by complex regulatory mechanisms. It is more reasonable to deduce that this NAC transcription factor influences processes promoting synthesis of photosynthetic machinery rather than degradation, since NAC expression decreased following leaf senescence. NAC expression was poorly correlated with Rubisco gene expression but relatively better correlated with chlorophyll content, suggesting NAC might regulate genes controlling chlorophyll synthesis.

The expression pattern of the closest barley orthologous gene of *TaNAC‐S*,* HvNAC015*, did not show any relation to senescence, with unchanged flag leaf expression throughout the post‐anthesis development period until complete senescence (Christiansen & Gregersen 2014). In addition, the closest rice orthologous gene of TaNAC‐S, *ONAC104*, is differentially expressed in response to infection with the rice tungro spherical virus (Nuruzzaman *et al*. 2010) and mild drought stress (Nuruzzaman *et al*. 2012), indicating a function in stress responses, although an opposite expression pattern for different stresses contradicts this. Since orthologous NAC transcription factors in wheat and rice have been shown to have diverse functions (Distelfeld *et al*. 2012), the rice expression pattern does not necessarily have relevance for the expression profile of the wheat gene. This also seems to be the case for the much more closely related barley *HvNAC015* gene. Many genes involved in senescence are also involved in stress responses (Nakashima *et al*. 2012); the response of the wheat NAC gene to different stressors requires further investigation.

### Leaf senescence and N remobilisation

Delaying senescence, and as a consequence restricting N remobilisation from leaf to grain, may lead to lower grain protein concentrations. For example, the grain protein concentration of a NAM‐B1 RNAi line with a stay‐green phenotype was significantly reduced (Uauy *et al*. 2006b). In this study, the total grain N content of transgenic lines with delayed senescence was not significantly different to that of the controls, while N concentrations were significantly enhanced in many of the transgenic lines. Generally, seed size and grain N concentration are negatively correlated, however our study indicated the grain N concentration of transgenic lines were significantly enhanced while seed size (in terms of 1000 grain weight) showed no significant difference to the control lines.

Similarly yield and grain N concentrations are usually negatively correlated. The stay‐green, NAC overexpressing plants achieved higher grain N concentrations than the wild type at similar grain yields (Fig. 6). This result contrasts with studies in which accelerated senescence resulted in increased grain protein content (Uauy *et al*. 2006b) and delayed senescence, which led to increased grain yield coupled with a decrease in grain protein content (Spano *et al*. 2003). However, Derkx *et al*. (2010, 2012) found that both fast‐senescing and stay‐green mutants of wheat had higher grain N concentrations than the wild type. This conflicting evidence suggests the involvement of different physiological mechanisms through which increased grain N concentrations may be achieved.

Increased grain N content may be accomplished through increased N uptake or improved partitioning of N to the grain through remobilisation. Most studies clearly point to the importance of remobilisation of N from the leaves to the grain. However, since greenness and leaf protein content are well correlated (Thomas *et al*. 2002), the stay‐green NAC overexpressing lines were likely to be impaired in N remobilisation, as demonstrated by the higher N content in the straw of all NAC overexpressing lines. Therefore, the higher grain N content is most likely explained as differences in post‐anthesis N uptake. This is consistent with the study of Bogard *et al*. (2010), who found that deviations from the grain protein concentration–grain yield relationship in wheat were consistently explained by differences in post‐anthesis N uptake.

It has been hypothesised that maintenance of N uptake during grain filling is determined by the carbon supply to the roots, and that this would result in a stay‐green phenotype due to less N remobilisation from the leaves (Rajcan & Tollenaar 1999). Such an increased carbon supply to the roots to increase N uptake offers a possible physiological explanation for how delayed leaf senescence can result in both lower aboveground biomass and higher grain N content.

In conclusion, TaNAC‐S may be a target for future wheat improvement strategies, since overexpression of this gene resulted in delayed senescence and higher grain N concentration.
